# Supplementary material for: Characterization of Two Parabacteroides distasonis Candidate Strains as New Live Biotherapeutics against Obesity
Source: Cells. 2023 Apr 26;12(9):1260. doi: 10.3390/cells12091260 (PMC10177344; doi:10.3390/cells12091260)
Supplement: Supplementary file 1 [file cells-12-01260-s001.zip › cells-2155481-supplementary.pdf]

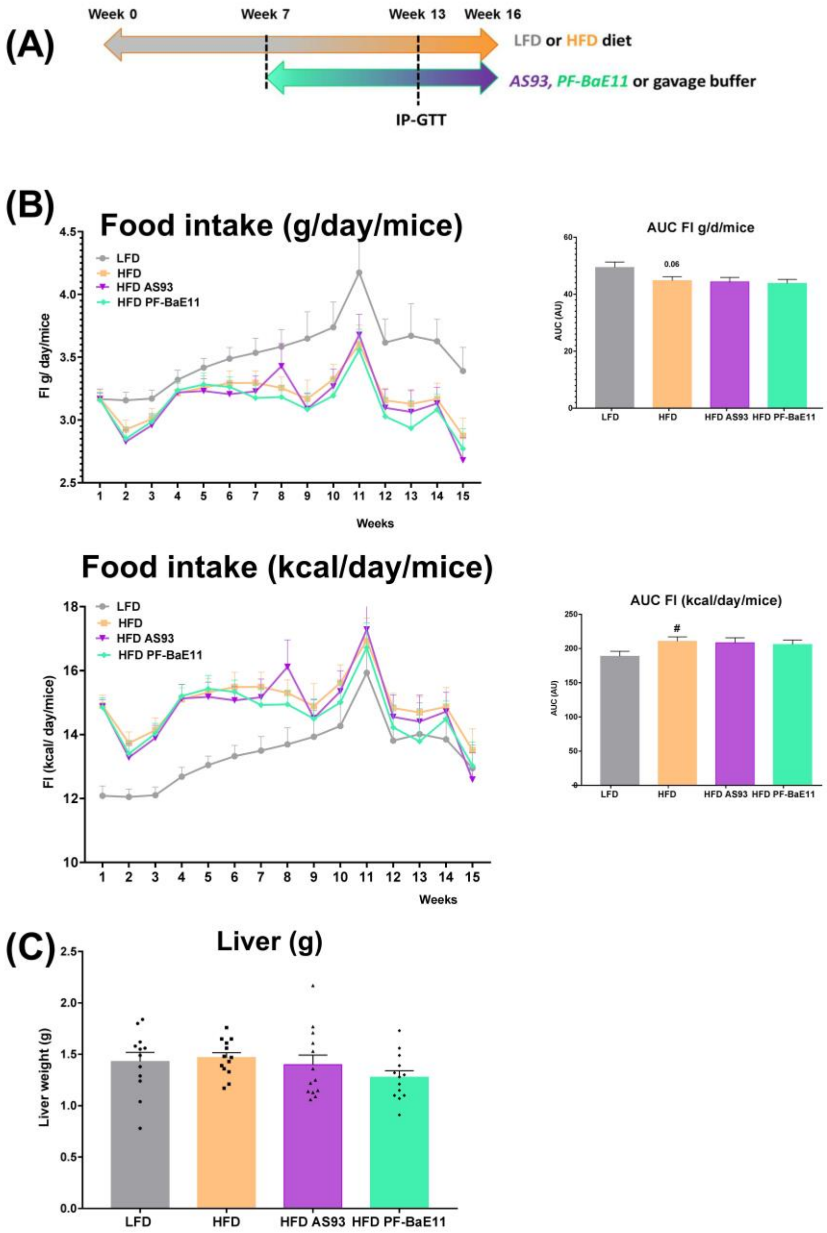

Figure S1. (**A**) Scheme of the experimental procedure used. Mice were fed with LFD or HFD. After 7 weeks, obese-HFD-fed mice were treated daily (5 days per week) by an intragastric administration (200 µl) of control gavage buffer or by a suspension of *P. distasonis* AS93 or PF-BaE11 (10^9 CFU/^{200} µL gavage buffer). Control LFD mice received an intragastric administration (200 µl) of control gavage buffer. Mice continued to be fed with respective diet and procedure was maintained for subsequent 9 weeks. IP-GTT was performed at 13 weeks post diet. (**B**) Daily food intake expressed in g/ day/ mice or in kcal/ day/mice and the corresponding AUC (in AU). (**C**) Mice liver weight in the 4 experimental groups expressed in g. Results are expressed as means ± SEM. # $p \leq 0.05$

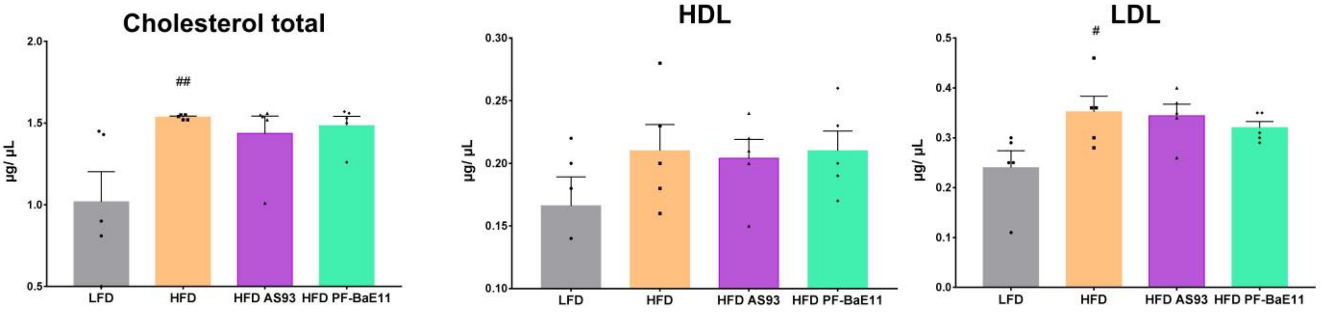

Figure S2. Levels of total cholesterol, HDL and VLDL/LDL determined using quantification kits provided by Abcam (Cambridge, UK). Results were expressed in µg/µL as mean ± SEM of 12 mice per groups. # $p \leq 0.05$, ## $p \leq 0.01$.
